# Conditional Reverse Tet-Transactivator Mouse Strains for the Efficient Induction of TRE-Regulated Transgenes in Mice

**DOI:** 10.1371/journal.pone.0095236

**Published:** 2014-04-17

**Authors:** Lukas E. Dow, Zeina Nasr, Michael Saborowski, Saya H. Ebbesen, Eusebio Manchado, Nilgun Tasdemir, Teresa Lee, Jerry Pelletier, Scott W. Lowe

**Affiliations:** 1 Cancer Biology and Genetics Program, Memorial Sloan Kettering Cancer Center, New York, New York, United States of America; 2 Department of Biochemistry, McGill University, Montreal, Quebec, Canada; 3 Watson School of Biological Sciences, Cold Spring Harbor Laboratory, Cold Spring Harbor, New York, United States of America; 4 The Rosalind and Morris Goodman Cancer Research Center, McGill University, Montreal, Quebec, Canada; 5 Howard Hughes Medical Institute, Memorial Sloan Kettering Cancer Center, New York, New York, United States of America; Baylor college of Medicine, United States of America

## Abstract

Tetracycline or doxycycline (dox)-regulated control of genetic elements allows inducible, reversible and tissue specific regulation of gene expression in mice. This approach provides a means to investigate protein function in specific cell lineages and at defined periods of development and disease. Efficient and stable regulation of cDNAs or non-coding elements (e.g. shRNAs) downstream of the tetracycline-regulated element (TRE) requires the robust expression of a tet-transactivator protein, commonly the reverse tet-transactivator, rtTA. Most rtTA strains rely on tissue specific promoters that often do not provide sufficient rtTA levels for optimal inducible expression. Here we describe the generation of two mouse strains that enable Cre-dependent, robust expression of rtTA3, providing tissue-restricted and consistent induction of *TRE*-controlled transgenes. We show that these transgenic strains can be effectively combined with established mouse models of disease, including both Cre/LoxP-based approaches and non Cre-dependent disease models. The integration of these new tools with established mouse models promises the development of more flexible genetic systems to uncover the mechanisms of development and disease pathogenesis.

## Introduction

Genetically engineered mouse models (GEMMs) are an invaluable tool to investigate the biology of development and disease in a mammalian organism. Since the development of the first knockout mouse almost 25 years ago, a wide variety of knockout, knock-in and conditional mutant strains have been developed to interrogate gene function [1]. In recent years, use of inducible promoters to control genetic elements in adult mice has become increasingly valuable, allowing regulated control of cDNAs and, more recently, shRNAs. In concept, these systems enable the timed expression or silencing of any gene, in any tissue and at any stage of development or disease progression. The application of such experimental tools promises a detailed understanding of the temporal requirements for gene function in specific tissues and provides an opportunity to genetically validate proposed therapeutic targets prior to drug development.

The tetracycline (tet) system is, by far, the most widely used inducible model in mice. It consists of two essential components: a tetracycline-responsive element (*TRE*) that regulates gene or shRNA expression, and a *trans*-acting, tet-sensitive, tet-transactivator (tTA) or reverse tet-transactivator (rtTA) protein [2]. tTA promotes gene expression from *TRE* promoters, but is inhibited in the presence of tet, or its more common analog, doxycycline (dox). Conversely, rtTA promotes dox-dependent gene induction. Early versions of the rtTA protein showed ‘leaky’ gene expression in the absence of dox, but newer variants such as rtTA^M2^ and rtTA3 [3], [4] show almost no dox-independent activity, and in the case of rtTA3, high sensitivity to low levels of dox. To date, more than 150 tTA/rtTA transgenic and knock-in strains have been developed to enable regulated gene expression in embryonic and adult tissues (http://www.tetsystems.com/fileadmin/tettransgenicrodents.pdf). As expression of the *TRE*-regulated cassette is dependent on both the presence of tet-transactivator and dox, induction can be ubiquitous or tissue specific (by controlling *tTA/rtTA* expression), inducible and reversible (by controlling dox exposure). Tissue specific *TRE* gene regulation is usually achieved by restricting the expression of tTA or rtTA to defined cell lineages using a tissue-specific promoter. Though convenient, this approach is absolutely dependent on robust expression of the tTA/rtTA. Moreover, cellular response downstream of transgene/shRNA induction may alter cell fate and compromise sustained *TRE*-regulated control. We recently described a new reverse tet-transactivator strain, *CAGs-rtTA3*, that shows stronger and more ubiquitous induction of *TRE*-regulated elements than any other existing strains we have tested [5]. Here we set out to develop a more flexible transgenic approach exploiting the strength of *TRE*-induction seen with *CAGs-rtTA3* and enabling tissue specific *TRE*-regulation.

## Results

### Conditional rtTA3 ES cell lines

**Figure 1:**
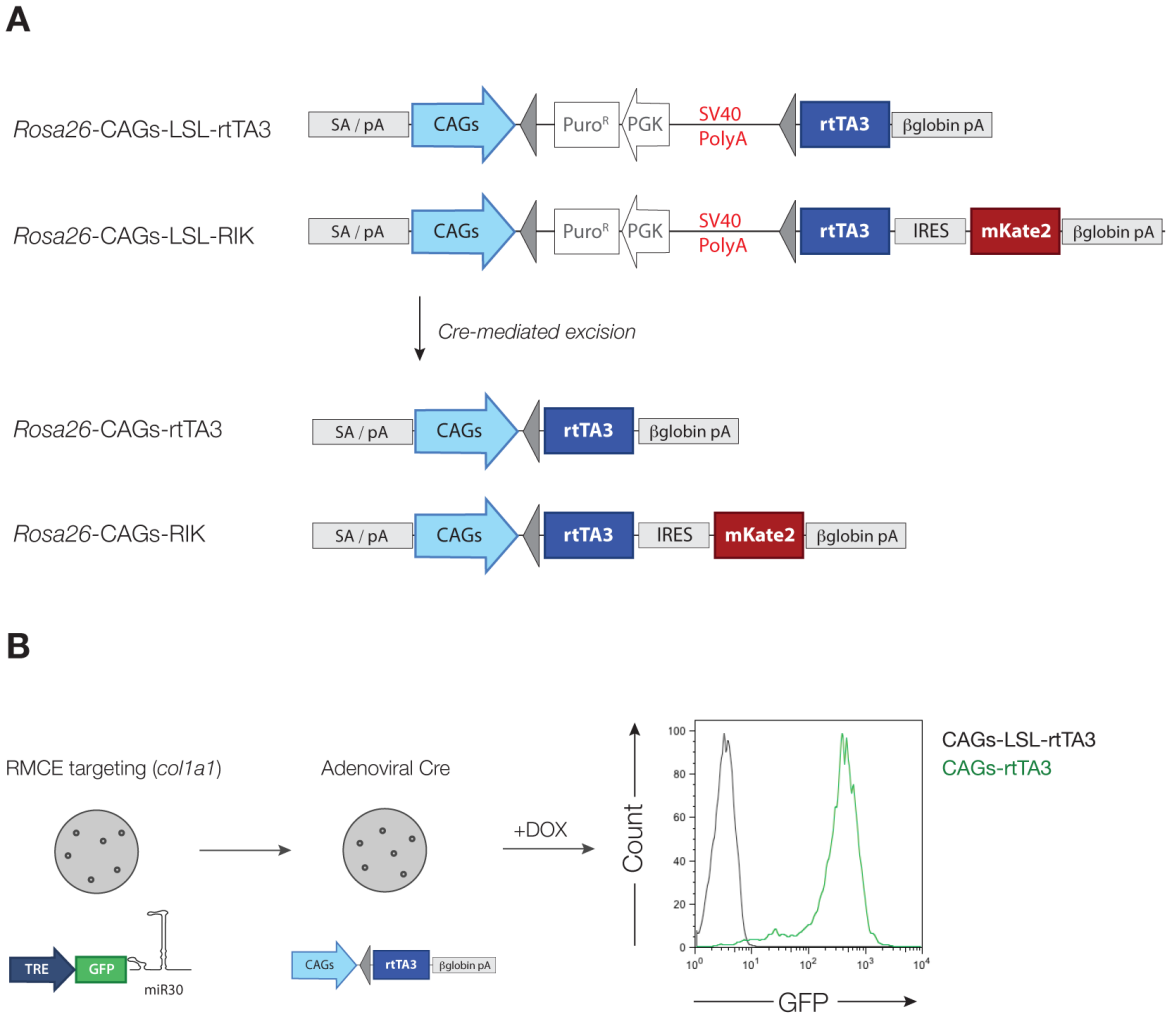
Generation of CAGs-LSL-rtTA3 and CAGs-LSL-RIK strains. **A**. Schematic representation of *CAGs-LSL-rtTA3* and *CAGs-LSL-rtTA3-IRES-mKate2 (RIK)* alleles targeted to the Rosa26 locus, prior to and following Cre-mediated recombination. **B**. Correctly targeted *CAGs-LSL-rtTA3* ESCs (Y1), were retargeted by recombinase mediated cassette exchange (RMCE) to introduce a *TRE-GFP-miR30* (TGM) construct to the *col1a1* recipient locus. Targeted cells were transduced with adenovirus expressing Cre and plated at low density to isolate individual clones. Clones were treated with doxycycline (1 ug/ml) for 2 days and analyzed by flow cytometry. Graph represents GFP fluorescence of TGM containing, dox-treated *CAGs-LSL-rtTA3* (black line) and recombined *CAGs-rtTA3* (green line) clones.

Conditional gene modification in mice is most commonly achieved using tissue specific Cre recombinase strains and hundreds of such mice have already been developed and tested; More than 300 Cre strains are available from the Jackson Laboratory. We reasoned that Cre-dependent expression of rtTA3 from the *CAGs* promoter would enable robust and tissue-specific *TRE*-transgene induction in almost any cell lineage. Thus, we modified our *CAGs-rtTA3* construct to contain a *LoxP*-flanked polyadenylation signal or LoxP-Stop-LoxP (LSL) cassette (*CAGs-LSL-rtTA3*). In addition, we cloned a variant that also carried the mKate2 far-red fluorescent gene [6] downstream of an *Internal ribosomal entry site* (*IRES*) (*CAGs-LSL-rtTA3-IRES-mKate2 – CAGs-LSL-RIK*) (Fig. 1A). In this context, mKate2 fluorescence serves as a reporter of Cre recombinase activity and rtTA3 expression. We cloned each construct into the *pRosa26-1* targeting backbone and transfected C10 ES cells [7]. Southern blot analysis of puromycin-selected clones identified correctly targeted clones for each construct (Fig. S1). In the case of *CAGs-LSL-RIK*, we identified one clone (designated D34), which showed homozygous targeting to the *Rosa26* locus. We further confirmed targeting on both copies of chromosome 6 in this ESC line by fluorescence in-situ hybridization (FISH) (Fig. S1C) and later, by breeding founder animals, which transmitted the allele to 100% of their progeny (not shown).

We first sought to confirm that both the *CAGs-LSL-rtTA3* (designated Y1) and *CAGs-LSL-RIK* (D34) cells showed robust expression of rtTA3 protein and therefore strong induction of *TRE*-regulated transgenes. To do this we took advantage of a recombinase-mediated cassette exchange (RMCE) ‘landing pad’ downstream of the *col1a1* locus in C10 ESCs [7]. This knock-in cassette allows efficient targeting of *TRE*-regulated (and other) transgenes, including cDNAs [8] and shRNAs [5]. We transfected Y1 and D34 cells with a *col1a1* RMCE targeting vector we previously described that expresses GFP and an shRNA directed against Renilla luciferase (*Ren.713*) downstream of *TRE* (*TG-Ren.713*). Throughout this study we use the *TG-Ren.713* transgene as a neutral fluorescent marker of dox-mediated induction. Following hygromycin selection, individual clones were expanded and transduced with a limiting titer of adenovirus expressing Cre recombinase to achieve recombination in 10–25% of cells. As expected, adenovirus transduced cells treated with doxycycline showed strong induction of GFP (Fig. S2). To confirm that single Cre-recombined clones showed uniform GFP induction we plated adenoviral treated Y1 cells at low density and isolated individual clones. 2/24 clones picked showed consistent and uniform, dox-dependent induction of GFP, whereas untreated cells showed no detectable GFP signal by flow cytometry (Fig. 1B).

To confirm the quality of Y1 and D34 ESCs for animal production, we generated wholly ESC-derived mice by tetraploid embryo complementation and bred multiple founder animals. Each cell line produced numerous viable and fertile mice that showed expected Mendelian transmission of the *Rosa26*-targeted allele (Table 1). Moreover, we also generated mice from both lines following *col1a1* re-targeting by RMCE (not shown), demonstrating that both Y1 and D34 are robust ESC lines that can serve as a platform for the production of conditional, TRE-inducible mice for analysis.

**Table 1:** Mendelian transmission of Rosa26-targted CAGs-rtTA3 transgenes.

Genotype	CAGs-LSL-rtTA3	CAGs-rtTA3	CAGs-LSL-RIK	CAGs-RIK
Transgene	219 (217)*	110 (124)	84 (85)	130 (137.5)
Wildtype	215 (217)	138 (124)	86 (85)	145 (137.5)
*p-value (two-tail, binomial test)*	0.89	0.09	0.94	0.40

*Numbers represent: observed (expected) from heterzygote x wild-type crosses.

### CAGs-rtTA3 and CAGs-RIK enable robust and widespread TRE-induction *in vivo*

We have previously noted that limited expression of rtTA from the endogenous *Rosa26* promoter (*R26-rtTA*) results in restricted induction of TRE-driven GFP in adult mouse tissues [5]. To test TRE-mediated induction from *CAGs-rtTA3* transgenes targeted to the *Rosa26* locus, we crossed each strain to a *CAGs-Cre* ‘deletor’ mouse that induces *LoxP* recombination at or before the two-cell stage [9]. Excision of the *LSL* cassette in the F1 progeny from each strain was confirmed by PCR and each of the recombined alleles could be propagated through breeding at Mendelian ratios, indicating there was no appreciable toxicity from the *CAGs*-driven expression of rtTA3 or mKate2 *in vivo* (Table 1).

**Figure 2:**
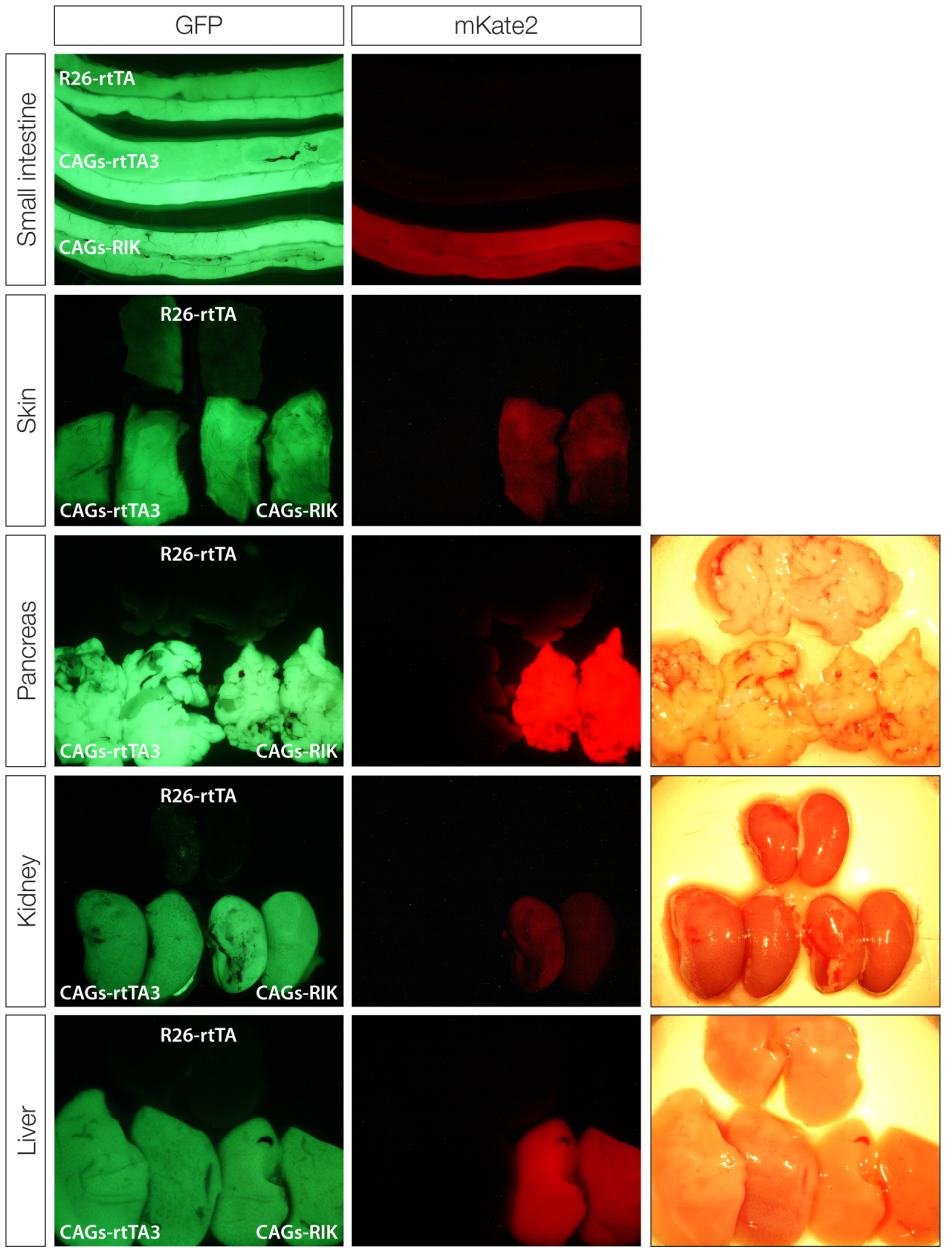
CAGs-rtTA3 and CAGs-RIK show strong expression in adult tissues. Whole mount epifluorescence images of small intestine, skin, pancreas kidney and liver from *R26-rtTA*, *CAGs-rtTA3* and *CAGs-RIK* transgenic animals (all containing *TG-Ren.713*). *R26-rtTA* shows strong expression in intestine and skin but weak or patchy expression in most other solid organs. *CAGs-rtTA3* and *CAGs-RIK* show almost identical expression patterns in adult mice. *CAGs-RIK* mice show strong and consistent expression of mKate2.

**Figure 3:**
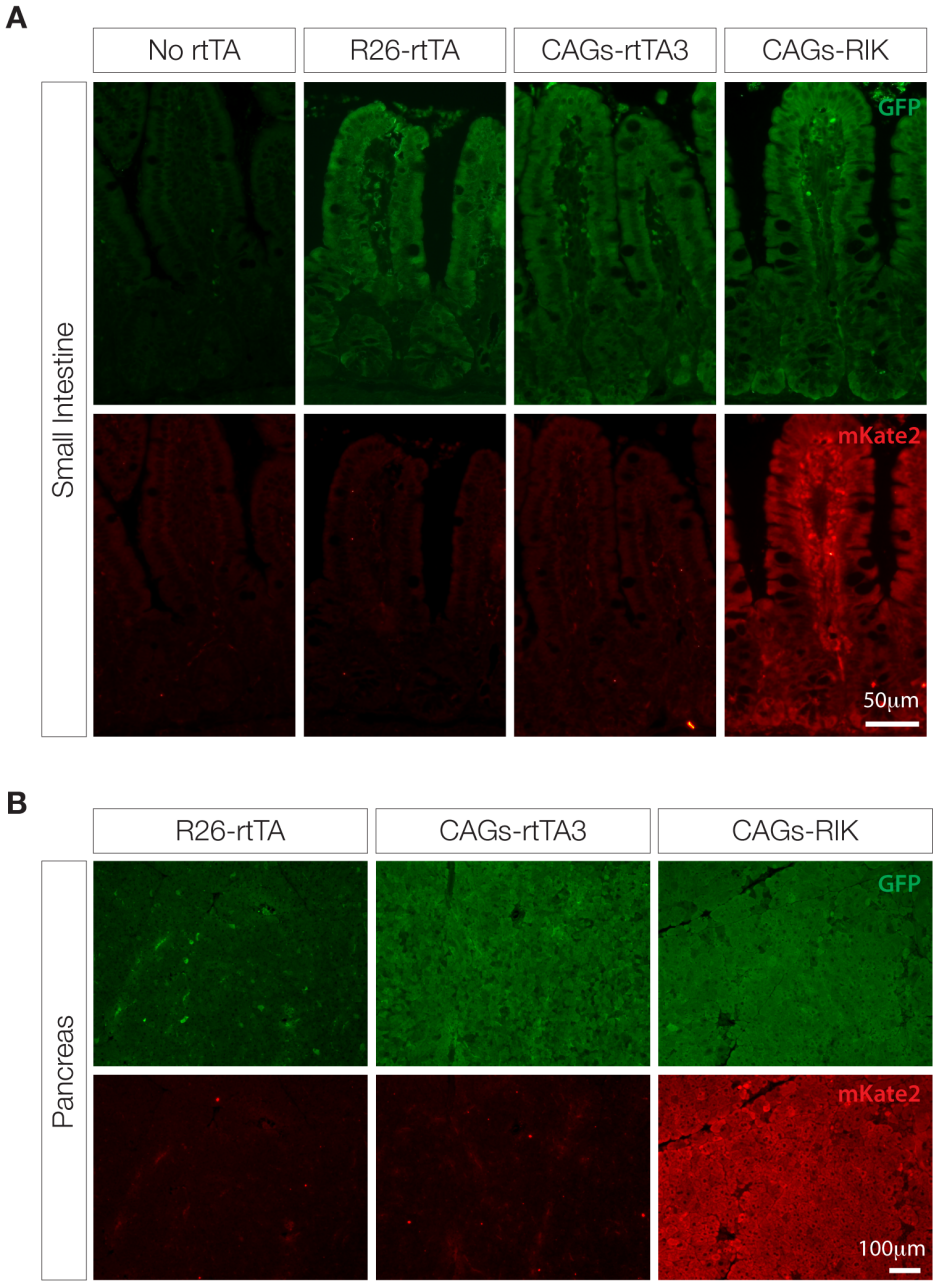
GFP induction and mKate2 expression is uniform in most organs of *CAGs-rtTA3* and *CAGs-RIK* mice. Immunofluorescence stains for GFP and mKate2 in the small intestine and pancreas of ‘no rtTA’, *R26-rtTA*, *CAGs-rtTA3* and *CAGs-RIK* mice following 1 week of doxycycline treatment. All rtTA strains show strong GFP induction in small intestine (**A**), but only *CAGs-rtTA3* and *CAGs-RIK* show robust and uniform GFP expression (and mKate2 for *RIK*) in the pancreatic acinar tissue (**B**).

**Figure 4:**
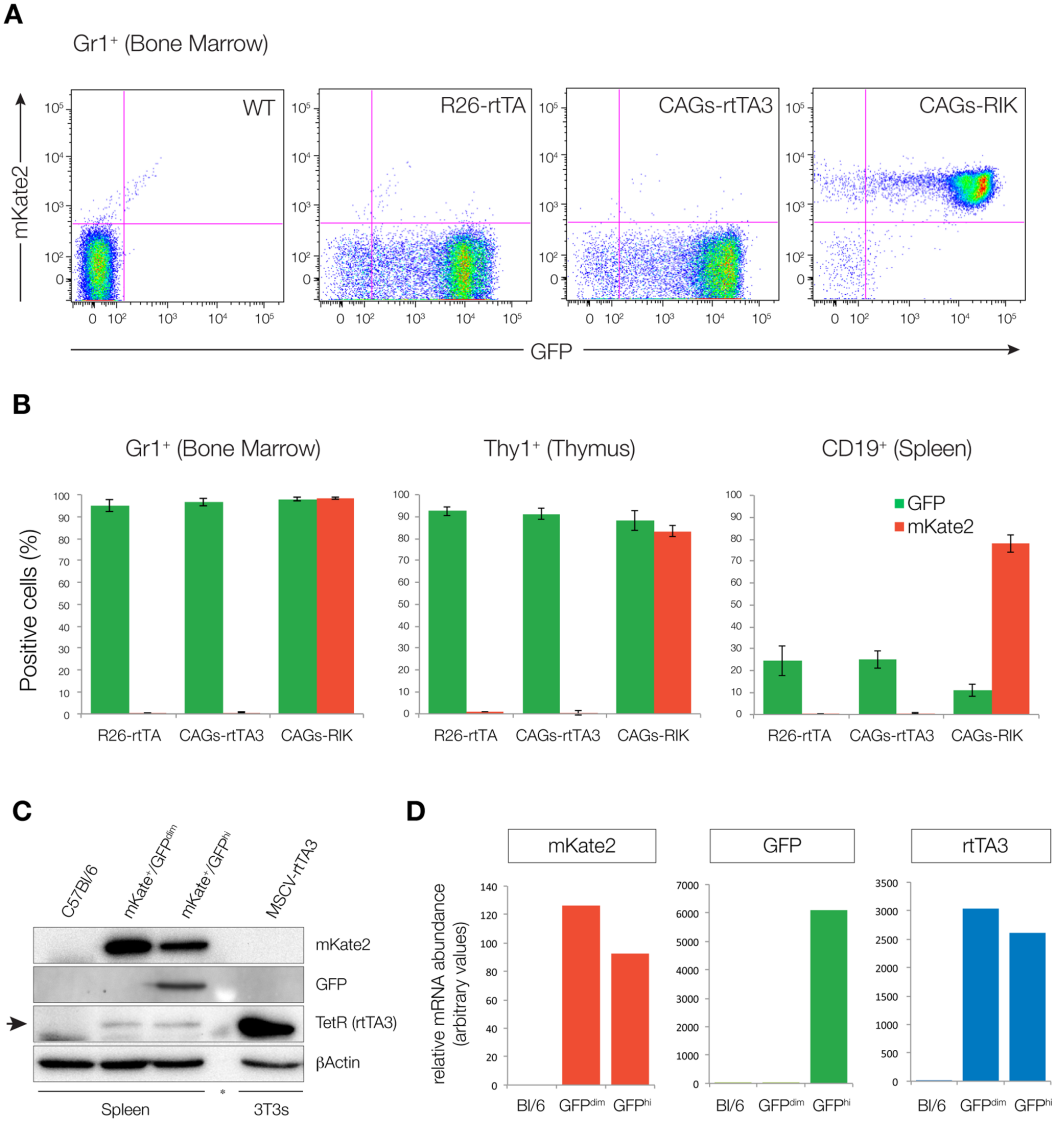
*CAGs-rtTA3* and *CAGs-RIK* enable GFP induction in myeloid and T lymphocyte lineages. **A**. Scatter plots representing GFP and mKate2 expression in Gr1 positive cells in the bone marrow of double transgenic (*rtTA/TGM*) animals following 1 week of doxycycline treatment (625 mg/kg in chow). **B**. Quantitation of GFP and mKate2 positive cells in Gr1, Thy1 and CD19 positive populations from the bone marrow, thymus and spleen respectively. Bars represent the mean percentage of GFP or mKate2 positive cells in each tissue, in 3 independent animals (per genotype) +/− SEM. **C**. Western blot of lysates from control (c57Bl/6), GFP negative and GFP positive splenocytes, indicating rtTA3, GFP and mKate2 expression in each population. Retrovirally transduced 3T3 cells serve as the positive control for rtTA3 expression. **D**. Graphs represent mRNA abundance in control (C57Bl/6), GFP negative and GFP positive splenocytes.

We next bred *CAGs-rtTA3* and *CAGs-RIK* mice to *TG-Ren.713* mice generated previously [5], allowing GFP fluorescence to serve as a neutral marker of TRE induction. Similar to what was observed in our transgenic *CAGs-rtTA3* strain, *Rosa26*-targeted *CAGs-rtTA3* and *CAGs-RIK* enabled robust TRE-driven GFP expression in most tissues, including both solid organs and hematopoietic cells (Figs. 2, 3, 4 and Fig. S3). *CAGs-rtTA3* and *CAGs-RIK* showed significantly higher GFP induction in skin, liver, kidney, pancreas, compared to *R26-rtTA* (Fig. 2), while all animals showed high-level induction in intestine and T cells (Figs. 3A and 4), as previously reported [5], [10]. Immunofluorescent staining for GFP and mKate2 revealed uniform and consistent staining in multiple cell types in each organ analyzed, compared to *R26-rtTA* that often showed absent or patchy expression in organs including pancreas and liver (Fig. 3B, Fig. S3B). As expected, *CAGs-RIK* mice showed uniform expression of mKate2 in all tissues examined. Interestingly, splenic B cells showed poor induction of the *TRE-GFP* transgene in all rtTA strains analyzed, including *CAGs-RIK*, despite relatively uniform mKate2. As mKate2 is expressed from the same polycistronic transcript as rtTA3, it is likely that these cells, which fail to induce high levels of GFP, contain abundant rtTA3 protein. To confirm this, we sorted mKate2+/GFP^dim^ and mKate2+/GFP^hi^ splenic cells from *CAGs-RIK/+;TG-Ren.713/+* mice treated with dox for one week, and assayed rtTA3 expression. Both QPCR and western blot analysis showed comparable levels of rtTA3 mRNA and protein in GFP^hi^ and GFP^dim^ cells (Fig. 4C,D), implying that the failure to induce GFP in some mKate2 positive cells is not due to reduced or absent rtTA3 expression, in contrast to previous observations with an independent CAGs-rtTA3 strain [10]. Although the mechanism underlying this observation is not known, it is possible that the *col1a1*-targeted, *TRE*-driven transgene is silenced or inaccessible in some cell types.

Importantly, both CAGs-driven strains showed almost identical GFP expression indicating that the presence of mKate2 in the *CAGs-RIK* strain did not alter rtTA3 levels. Together this data confirms that the CAGs promoter (at the *Rosa26* locus) can drive strong and widespread expression of rtTA3, allowing dox-dependent *TRE* induction in almost all tissues. Importantly, this implies that in combination with an appropriate Cre driver, both *CAGs-LSL-rtTA3* and *CAGs-LSL-RIK* strains can provide robust, tissue and/or cell-type specific TRE-mediated gene expression.

### Mosaic TRE-induction through adenoviral Cre delivery

**Figure 5:**
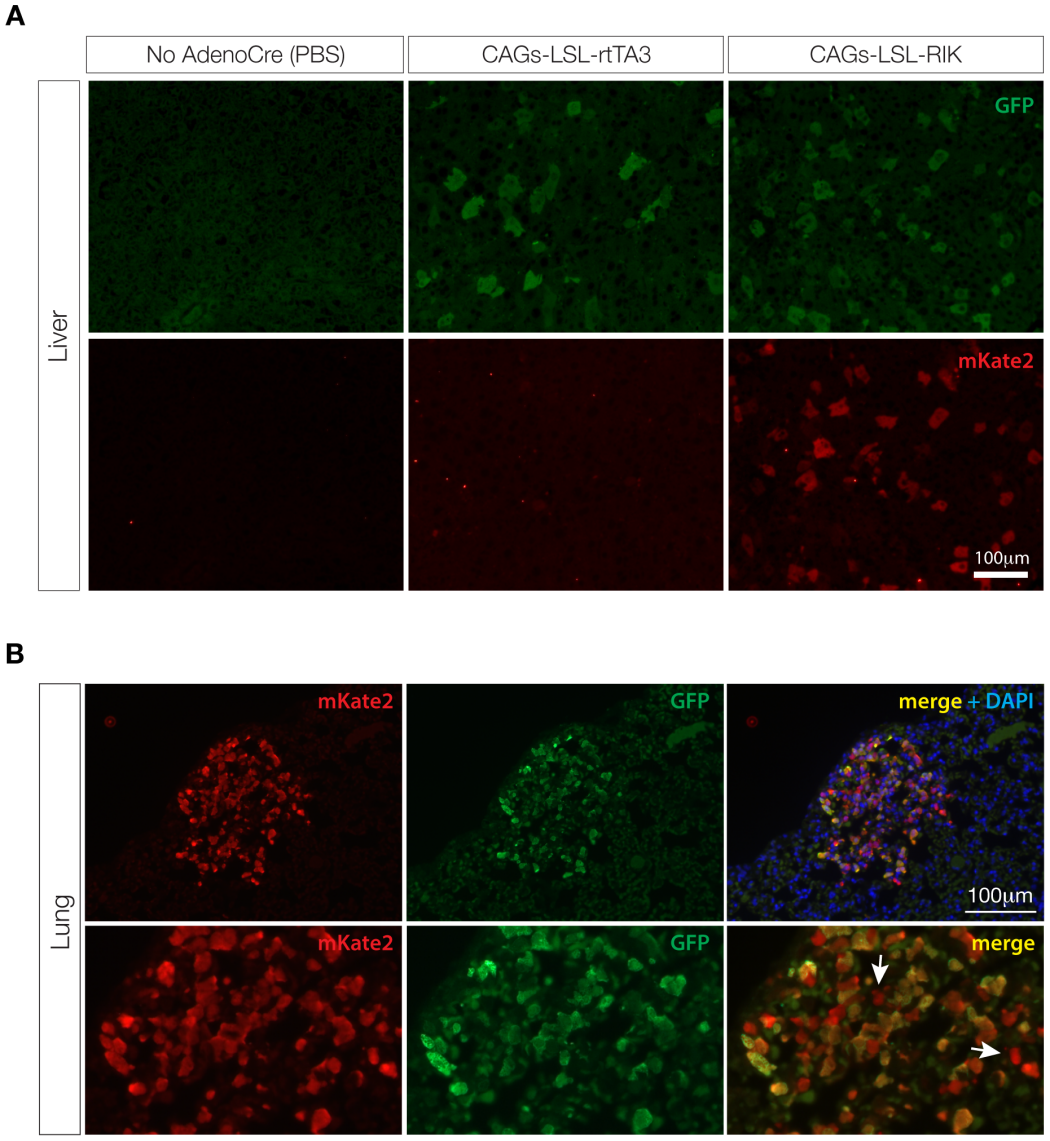
Adenoviral Cre induces mosaic activation of rtTA and GFP induction in *CAGs-LSL-rtTA3* and *CAGs-LSL-RIK* animals. **A**. Immunofluorescent stains for GFP and mKate2 in liver sections of *TG-Ren.713;CAGs-LSL-rtTA3* and *TG-Ren.713;CAGs-LSL-RIK* mice 1 week following intravenous injection of Adenoviral Cre (5×10^8^ PFU) or PBS (*CAGs-LSL-RIK* only – left panel) and dox treatment. Double transgenic mice exposed to AdenoCre show mosaic expression of GFP (*CAGs-LSL-rtTA3*) or GFP and mKate2 (*CAGs-LSL-RIK*). No GFP of mKate2 expression was observed in animals not exposed to Cre. **B**. Immunofluorescent stains for GFP and mKate2 in lung sections of triple transgenic mice (*CAGs-LSL-rtTA3 or RIK;TG-Ren.713;LSL-Kras^G12D^*). Kras^G12D^-induced lung adenomas show strong expression of GFP and mKate2. Lowe panel: higher magnification of the lesion. White arrows indicate rare cells that show mKate2, but not GFP expression.

Tissue restricted Cre recombinase expression can be achieved in mice through the delivery of virus (adeno- or lentivirus) to specific organs. Intravenous (tail-vein) injection of adenovirus results in almost exclusive transduction of liver hepatocytes. As a first step to evaluate Cre-mediated, tissue specific TRE-induction we injected *CAGs-LSL-rtTA3* and *CAGs-LSL-RIK* mice (also carrying *TG-Ren.713*) with Adenoviral-Cre (AdenoCre) and treated mice with dox for one week. As expected, AdenoCre, dox-treated *CAGs-LSL-rtTA3* and *CAGs-LSL-RIK* mice showed mosaic expression of GFP (and mKate2 in *CAGs-LSL-RIK* animals) in the liver (Fig. 5A). Importantly, we have never observed GFP signal in the absence of mKate2, suggesting mKate2 is a reliable indicator of rtTA3 expression, and no GFP was detected in any of the organs from dox-treated animals that were not exposed to Cre, indicating very low or no leaky expression of the rtTA3 transgene in adult mice.

Restricted delivery of AdenoCre to the mouse trachea allows mosaic Cre-mediated recombination in the lung epithelium [11]. We have previously shown that the Clara-cell Secretory Protein (CCSP)-rtTA transgenic strain can drive lung-specific expression of GFP-linked shRNAs in combination with AdenoCre-induced Kras^G12D^ [5]. To assess whether our Cre-dependent rtTA3 alleles would similarly allow dox-dependent shRNA induction in Kras^G12D^ expressing lung epithelium we treated *LSL-Kras^G12D/+^;TG-Ren.713/+;CAGs-LSL-RIK/+* mice with AdenoCre (5×10^6^ PFU) via intratracheal injection and treated with dox for one week prior to analysis. Three months following AdenoCre treatment animals showed small Kras^G12D^-driven adenomas throughout the lung epithelium (Fig. 5B, Fig. S4) consistent with previous reports [12]. In almost all cases, Kras^G12D^-driven adenomas were GFP and mKate2 positive, indicating strong expression of rtTA3 and TRE-regulated shRNAs in these lesions. We observed only a very small proportion of individual mKate2 positive cells that did not express GFP (Fig. 5B, white arrows). In addition, in some lesions we noted some small areas of adenomas that were mKate2 negative (Fig. S4), implying activation of *LSL-Kras^G12D^* without *CAGs-LSL-RIK* recombination. It was not possible to determine whether there were also cases of *CAGs-LSL-RIK* recombination without Kras^G12D^ activation, as these events would not expand into adenomas. In all, this work demonstrates that both *CAGs-LSL-rtTA3* and *CAGs-LSL-RIK* allow robust and tissue-specific expression of rtTA3 in adult mice following restricted Cre exposure.

### Tissue specific TRE-induction in transgenic Cre tumor models

In principle, integration of the Cre-conditional rtTA approach described above into existing disease models would enable a more precise investigation of gene function in complex genetic backgrounds. To test this we incorporated the *CAGs-LSL-RIK* into two well-established models, specifically, a Cre-dependent, *LSL-Kras^G12D^*-driven pancreatic cancer model [13] and Cre-independent, *MMTV-ErbB2* driven mammary cancer model [14].

**Figure 6:**
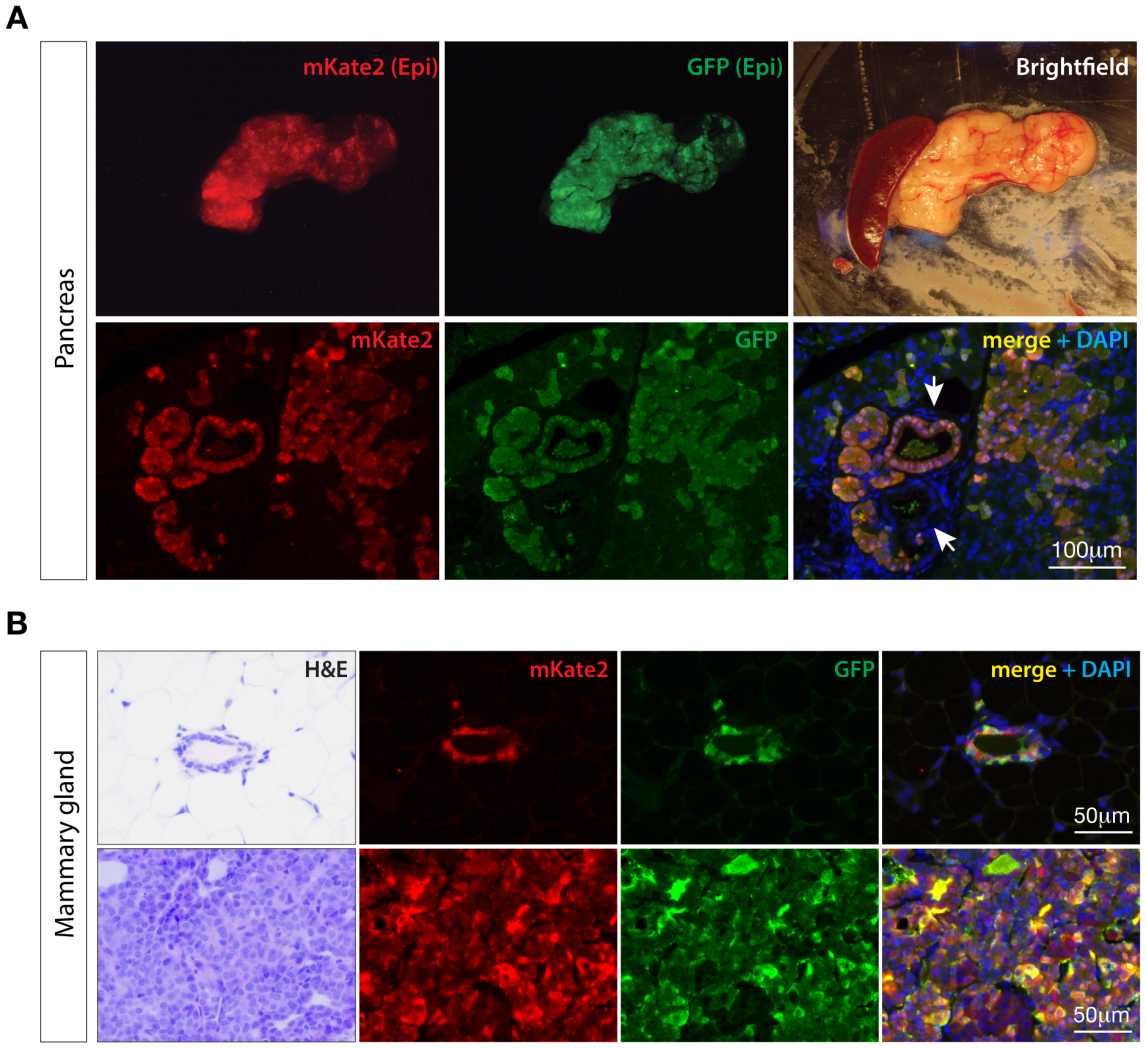
*CAGs-LSL-RIK* enables tissue-restricted expression of *TRE*-transgenes in transgenic models of disease. **A**. Whole mount epifluorescence (top panel) and immunofluorescence images from a quadruple transgenic (*CAGs-LSL-RIK;TG-Ren.713;LSL-Kras^G12D^;Pdx1-Cre*) animal, showing induction of GFP and mKate2 in both normal acinar tissue and pre-neoplastic, Kras^G12D^-induced PanIN lesions (top arrow). As observed in AdenoCre treated lungs, some PanIN lesions did not show GFP or mKate2 staining suggesting incomplete LSL excision in a small proportion of cells. **B**. Immunofluorescent stains for GFP and mKate2 in mammary tissue of *CAGs-LSL-RIK;TG-Ren.713;MMTV-Neu;WAP-Cre* transgenic mice treated with dox.

Kras (codon 12 or 13) mutation is considered an initiating and driving event in most pancreatic ductal adenocarcinomas. In the mouse, pancreatic restricted expression of Kras^G12D^, via a pancreas specific Cre such as *Pdx1-Cre*, leads to preneoplastic PanIN lesions [13]. We generated quadruple transgenic mice carrying *LSL-Kras^G12D/+^;Pdx1-Cre/+;CAGs-LSL-RIK/+;TG-Ren.713/+* and treated them with doxycycline until 3 months of age. At this time the pancreas showed mosaic expression of mKate2 and GFP due to mosaic expression of the *Pdx1-Cre* allele. As expected, histology of the pancreas in 3-month old transgenic animals showed both normal acinar tissue and the development of acinar-to-ductal metaplasia and early PanIN lesions, as previously described [13]. The majority of PanIN lesions showed strong GFP and mKate2 expression confirming robust induction of rtTA3 and the GFP-linked shRNA. As noted following AdenoCre treatment in the lung, we observed some small regions of ADM/PanIN lesions that did not express mKate2 or GFP (Fig. 6A, white arrows). We have now used the combination of Cre-driven pancreatic lesions with inducible shRNAs to investigate the genetic requirements underlying the initiation and progression of Kras-driven pancreas cancer [15].

Overexpression of HER2/Neu (encoded by the *ERBB2* gene) is a common feature of human breast cancer. In the mouse, this event can be mimicked via expression of a mutant rat ortholog *neu^V664E^* allele downstream of a mouse mammary tumor virus (MMTV) promoter [14]. The *MMTV-neu^V664E^* model has been used extensively to investigate the genetics and physiology of breast cancer progression. Expression of neu^V664E^ in this model is not dependent on the activity of Cre recombinase, therefore we asked whether our Cre-dependent *CAGs-LSL-RIK* allele could be used effectively in combination with *MMTV-neu^V664E^* to express inducible shRNAs in the mammary gland and drive tumorigenesis. For this, we generated female *MMTV-neu^V664E/+^;CAGs-LSL-RIK/+;TG-Ren.713/+* animals also carrying the murine *whey acidic protein* gene promoter (WAP)-driven Cre transgene which responds to lactogenic hormones [16]. Parous females nursed litters for 3 weeks to induce expression of *WAP-Cre* and were treated with doxycycline to induce shRNA expression. These mice showed luminal epithelial expression of mKate2 and GFP (Fig. 6B, top panel), and developed neu^V664E^-driven GFP and mKate2 positive tumors at a median latency of approximately 160 days post partum (Fig. 6B, lower panel). Although not all tumors from *TG-Ren.713* mice showed mKate2/GFP expression due to an incomplete overlap of expression between the MMTV and *WAP* promoters, this novel multi-allelic system has proven a powerful platform for the study of tumor suppressor gene function in the context of HER2-driven breast cancer (SHE and SWL, *unpublished data*).

## Discussion

Spatial and temporal control of gene expression provides a means to understand the contribution of genetic disruptions to disease progression and offers a setting to interrogate the role of individual genes in disease maintenance. Here we describe the generation and characterization of two novel ESC lines and mouse strains that enable Cre-dependent, robust expression of the reverse tet-transactivator (rtTA3) and thus, tissue-restricted induction of TRE-controlled transgenes. Further, we show that these transgenic strains can be effectively combined with established mouse models of disease, including Cre/LoxP-based approaches and Cre-independent model systems. The integration of established models of disease with the flexibility of inducible and reversible gene regulation will allow a more detailed interrogation of the underpinnings of disease pathogenesis and evolution. For instance, model systems that incorporate constitutive and inducible genetic alterations with inducible and reversible gene silencing (or overexpression) offer the unique opportunity to investigate how the temporal order of events determines disease progression and whether those events are required for disease maintenance. Such work will ultimately lead to better understanding of disease and the development of more accurate and effective preclinical models.

In contrast to strategies that rely on tet-transactivators driven by tissue specific promoters (http://www.tetsystems.com/fileadmin/tettransgenicrodents.pdf) our approach integrates the use of established tissue-restricted Cre recombinase strains to initiate *TRE* induction. Previous studies have reported the generation of Cre-dependent tTA and rtTA strains driven by the endogenous *Rosa26* promoter [17]–[19]. We have previously shown that *Rosa26* promoter activity can vary significantly in different tissues of adult mice, resulting in sub-optimal *TRE* induction in a range of cell types [5]. In contrast, the synthetic, ubiquitous *CAGs* promoter provides robust *TRE* induction in most adult tissues (Fig. 2). Thus, given an appropriate Cre driver, *CAGs-LSL-rtTA3* and *CAGs-LSL-RIK* provide potent, doxycycline-dependent transgene/shRNA expression in most tissues accessible to doxycycline. In addition, as the *CAGs* promoter drives robust expression of rtTA3 in many different cell lineages and at different stages of differentiation, *CAGs-LSL-rtTA3* strains offer a significant advantage over *tTA/rtTA* strains that depend on lineage specific promoters, such as those expressed only in stem or progenitor cells. In this regard, we have observed that transient induction of Cre recombinase activity in stem cells of the intestine using *Lgr5-GFP-IRES-CreER*, promotes long-lived rtTA3 expression (and GFP induction) in individual crypts and villi of the small and large intestine (LED and SWL, unpublished data).

During our analysis of multi-allelic animals carrying *LSL-Kras^G12D^* and *CAGs-LSL-RIK*, we noted some areas of tissue that showed characteristic Kras^G12D^-induced changes (lung adenomas or PanINs), which did not show expression of mKate2, suggesting Cre-induced recombination of only a subset of ‘floxed’ genes in the genome. Because Kras^G12D^-driven phenotypes are not 100% penetrant in either tissue, we have not been able to measure the frequency of cells that show recombination only at the *CAG-LSL-RIK* allele and not *Kras^G12D^*. The reasons behind this incomplete recombination are not clear but may reflect the regions surrounding the *LoxP* sites. Of note, we have observed increased recombination efficiency of the *CAGs-LSL-rtTA3* allele compared to the *CAGs-LSL-RIK* strain in cases of low or transient Cre expression; the two alleles vary slightly in sequences close to the 3′ LoxP site due to alternate cloning strategies. It is likely that incomplete *LoxP* recombination is a feature of many complex, Cre-dependent models, but it goes undetected due to a lack of reporter-based approaches. The presence of mKate2 as a Cre reporter in *CAGs-LSL-RIK* provides a means to clearly identify cells and tissues that express rtTA3 and are capable of inducing shRNA or transgene expression, irrespective of fluorescent tags linked to such transgenes (e.g. GFP). Thus, it enables tracking and/or prospective isolation of Cre-recombined cells prior to induction of, and post-withdrawal of, *TRE*-driven transgene/shRNA expression.

We and others recently described a conceptually new approach to complex mouse modeling, based on the derivation and manipulation of conditional, multi-allelic embryonic stem cells (ESCs), which we use to generate tailored genetic models for analysis [1], [5], [20]. Such ESC-GEMMs provide a means to rapidly interrogate gene function in genetically complex animal models in a fraction of the time required for traditional breeding. During the genesis of the two transgenic strains described here we generated ESC lines carrying *CAGs-LSL-rtTA3* (Y1) or *CAGs-LSL-RIK* (D34) as well as the *col1a1* homing cassette for RMCE. Thus Y1 and D34 cells could be employed by investigators wanting to fast track analysis of a gene or genes in a setting where Cre recombinase is delivered extrinsically (i.e. intratracheal or intravenous injection) or used as a base ESC line for the introduction of additional genetic manipulations, such as the incorporation of tissue specific Cre knock-in alleles. Alternately these alleles could be incorporated into ESC-GEMMs through re-derivation of new ESC lines. In fact, we have recently validated the use of this approach by producing a number of Kras^G12D^-based pancreatic cancer models, using *CAGs-LSL-RIK* to drive pancreas-specific expression of positive and negative regulators of tumor initiation and progression [15].

Together, the ESCs and mouse strains described here bolster the already impressive arsenal of *in vivo*, tet-based systems for manipulation of gene expression by providing robust tools for tissue-restricted induction of transgenes and shRNAs. Integrating these new strains with existing mouse modeling platforms promises to provide a wealth of new discoveries by unearthing the details of gene function in all stages of development and throughout the pathogenesis of disease.

## Materials and Methods

### ES cell targeting

All ES cells were maintained on irradiated feeders in M15 media containing LIF as previously outlined [21]. Targeting vectors were linearized using a unique PmeI site introduced downstream of the Diphtheria Toxin A (DTA) expression cassette. C2 ES cells (1×10^7^) were electroporated with 50 ug linearized targeting vector using a BioRad Gene Pulser and plated in M15 media as previously described [21]. Two days following transfections cells were treated with media containing 1 ug/ml puromycin and individual surviving clones were picked after 9–10 days of selection. Two days after clones were picked puromycin was removed from the media and cells were cultures in standard M15 thereafter. For Southern blots, genomic DNA from individual ES cell clones was digested overnight in either EcoRI or EcoRV/BglII (See Fig. S1).

### Animal husbandry

ES cell-derived mice were produced by tetraploid complementation as has been described elsewhere (Zhao, Nat Prot 2010). For removal of the ‘LSL cassette’ *in vivo*, CAGs-LSL-rtTA3 and CAGs-LSL-RIK (*R26Sor^tm1(CAGs-LSL-rtTA3)Slo^ and R26Sor^tm2(CAGs-LSL-RIK)Slo^*) mice were crossed to the CAGs-Cre transgenic strain and F1 progeny were genotyped for LoxP recombination using specific primers (see Table S1). TG-Ren.713 [5], WAP-Cre (Wagner et al, 1997), MMTV-Neu (Muller et al, 1988), LSL-KrasG12D [12] and Pdx1-Cre [13] mice have all been previously described. For mammary gland experiments, female mice were bred at 7 weeks and doxycycline feed was administered from date of litter birth. Litters were nursed for 3 weeks to induce WAP promoter activity. Parous mice monitored weekly for tumor formation by physical palpation. See Table S1 for genotyping information on CAGs-LSL-rtTA3 and CAGs-LSL-RIK strains.

### Ethics statement

Production of mice and all treatments described were approved by the Institutional Animal Care and Use Committee (IACUC) at McGill University (Montreal, Canada) or Memorial Sloan Kettering Cancer Center (NY) under protocol numbers: 2001–4751 (McGill), 11-06-012, 11-06-015 and 12-04-006 (MSKCC).

### Treatments

Doxycycline was administered via food pellets (625 mg/kg) (Harlan Teklad). Adenovirus expressing Cre recombinase (AdenoCre) was purchased from The University of Iowa Gene transfer Core. *For adenoviral delivery to the liver*: 5×10^8^ PFU AdenoCre/mouse was injected intravenously via the tail vein. *For adenoviral delivery to the lung*: 6–10 week-old mice were anesthetized by i.p. injection of ketamine 80 mg/kg, xylazine 10 mg/kg [11] and treated once by intratracheal instillation of 5×10^6^ PFU AdenoCre/mouse. Three months following AdenoCre treatment, lungs were collected, fixed and analyzed by immunofluorescence or immunohistochemistry.

### Protein and RNA analysis

Protein lysates were prepared in RIPA buffer and quantified by Lowry assay (BioRad). Western blots were probed with antibodies against: TetR (rtTA3) (1∶1000, mouse monoclonal Clone 9G9, Clontech #631131), GFP (1∶2000, chicken polyclonal, Abcam #ab13970), tRFP (mKate2) (1∶2000, rabbit polyclonal, Evrogen #B00201) and β-Actin-HRP (1∶5000, mouse monoclonal AC15 clone, Sigma #A3854). RNA was prepared from sorted cells by Trizol extraction and column purification. cDNA was prepared from 1 µg total RNA using Taqman reverse transcription kit (Applied Biosystems, #N808-0234) with random hexamers. Quantitative PCR detection was performed using SYBR green reagents (Applied Biosystems) using primers specific to ***rtTA3***: F: 5′-CAATGGTGTCGGTATCGAAG-3′, R: 5′-CTTGTTCTTCACGTGCCAGT-3′; ***mKate2***: F: GGTGAGCGAGCTGATTAAGG-3′ and R: 5′-TTTTGCTGCCGTACATGAAG-3′; and ***GFP***: F: 5′-ATCGACTTCAAGGAGGACGGCA-3′ and R: 5′-CGTTCTTCTGCTTGTCGGCCAT-3′.

### Immunophenotyping

Immunostaining and FACS analysis for blood lineages were performed as previously described [22]. Briefly, single cell suspensions from whole bone marrow, spleen and thymus were immunostained for CD45.2 (APC-conjugate, BD #559864) and cell lineage markers: Gr1 (Pacific blue, BioLegend #108430), CD19 (Pacific Blue, BioLegend #115526) or Thy1 (Pacific blue, BioLegend #105324) and the percentage of GFP and/or mKate2 expressing cells were calculated within these specific lineages populations. Data was collected on an LSR-II flow cytometer (BD BioSciences) and analyzed using FlowJo software (Tree Star).

### Immunohistochemistry

Tissue, fixed in 10% neutral buffered formalin for 24 hours, was embedded in paraffin and sectioned by IDEXX RADIL (Columbia, MO). Sections were rehydrated and unmasked (antigen retrieval) by heat treatment for 5 mins in a pressure cooker in 10 mM Tris/1 mM EDTA buffer (pH 9) containing 0.05% Tween 20. For immunohistochemistry, sections were treated with 3% H_2_O_2_ for 10 mins and blocked in TBS containing 1% BSA. For immunofluorescence, sections were not treated with peroxidase. Primary antibodies, incubated at 4C overnight in blocking buffer, were: chicken anti-GFP (1∶500, #ab13970), rabbit anti-tRFP (1∶2000, Evrogen, #AB232) and rabbit anti-ki67 (1∶100, Sp6 clone, Abcam #ab16667). For immunohistochemistry, sections were incubated with anti-rabbit ImmPRESS reagent (Vector Laboratories, #MP7401) and developed using ImmPACT DAB (Vector Laboratories, #SK4105) according to the manufacturer instructions. For immunofluorescent stains, secondary antibodies were applied for 1 hour at room temp in TBS in the dark, washed twice with TBS, counterstained for 5 mins with DAPI and mounted in ProLong Gold (Life Technologies, #P36930). Secondary antibodies used were: anti-chicken 488 (1∶500, DyLight IgG, #ab96947) and anti-rabbit 568 (1∶500, Molecular Probes, #a11036). Images of fluorescent and IHC stained sections were acquired on a Zeiss Axioscope Imager Z.1 using a 10x (Zeiss NA 0.3) or 20x (Zeiss NA 0.17) objective and an ORCA/ER CCD camera (Hamamatsu Photonics, Hamamatsu, Japan). Raw.tif files were processed using Photoshop CS5 software (Adobe Systems Inc., San Jose, CA) to adjust levels and/or apply false coloring.

## Supporting Information

Figure S1 **Targeting** ***CAGs-LSL-rtTA3*** **to the** ***Rosa26*** **locus.** **A**. Schematic of the *Rosa26* locus before and after recombination of the *CAGs-LSL-rtTA3* or *CAGs-LSL-RIK* targeting vector. Key restriction sites used for clone identification by Southern blot are indicated. Sizes of each predicted fragment are also shown and a solid black line highlights the position of the Southern probe. **B**. Southern blot images showing identification of Y1 (2.3 kb band) and D34 (4.8 kb band) clones, following EcoRV/BglII and EcoRI digests, respectively. **C**. Fluorescence in situ hybridization on a metaphase spread from D34 ES cells using the CAGs-LSL-RIK fragment as a probe, showing homozygous targeting of *CAGs-LSL-RIK* to Chromosome 6.
(TIF)

Figure S2 **GFP induction following Adenoviral Cre transduction in targeted Y1 and D34 ESCs.** Y1 and D34 ESCs carrying *TG-Ren.713* at the *col1a1* locus were transduced with adenovirus expressing Cre (Cre, green line) or not transduced (no Cre, black line), treated with doxycycline (1 ug/ml) for 2 days and analyzed by flow cytometry. Graphs represent bulk population of transduced cells (not single clones). Bulk populations were single cell cloned to assess the uniformity of GFP induction in the presence of constitutive rtTA3 expression (see Fig. 1B).
(TIF)

Figure S3 **GFP induction and mKate2 expression in large intestine and liver.** Immunofluorescence stains for GFP and mKate2 in the large intestine and liver of ‘no rtTA’, *R26-rtTA*, *CAGs-rtTA3* and *CAGs-RIK* mice following 1 week of doxycycline treatment. All rtTA strains show strong GFP induction in large intestine (**A**), but only *CAGs-rtTA3* and *CAGs-RIK* show robust and uniform GFP expression (and mKate2 for *RIK*) in the liver tissue (**B**).
(TIF)

Figure S4 **Mosaic mKate2 expression in a proportion of lung adenomas.** Immunohistochemical stains for mKate2 and Ki67 in lung sections of double transgenic mice (*CAGs-LSL-RIK;LSL-Kras^G12D^*) treated with intratracheal Adenoviral Cre (AdenoCre) or vehicle (Tris-HCl). 12 weeks following Cre delivery LSL-KrasG12D mice show small, moderately proliferative adenomas. Some adenomas show uniform mKate2 staining (top panel of ‘AdenoCre’), while a subset showed both positive and negative mKate2 cells (arrows) suggesting Cre-driven activated Kras^G12D^ but not rtTA3-IRES-mKate2. Adenoma area highlighted by dotted line.
(TIF)

Table S1 **Genotyping primers.** Primer sequences and expected PCR product sizes for genotyping *Rosa26*-targeted CAGs-rtTA3 strains.
(DOCX)
