# Supplementary figures and images for: Conditional Reverse Tet-Transactivator Mouse Strains for the Efficient Induction of TRE-Regulated Transgenes in Mice
Source: PLoS One. 2014 Apr 17;9(4):e95236. doi: 10.1371/journal.pone.0095236 (PMC3990578; doi:10.1371/journal.pone.0095236)

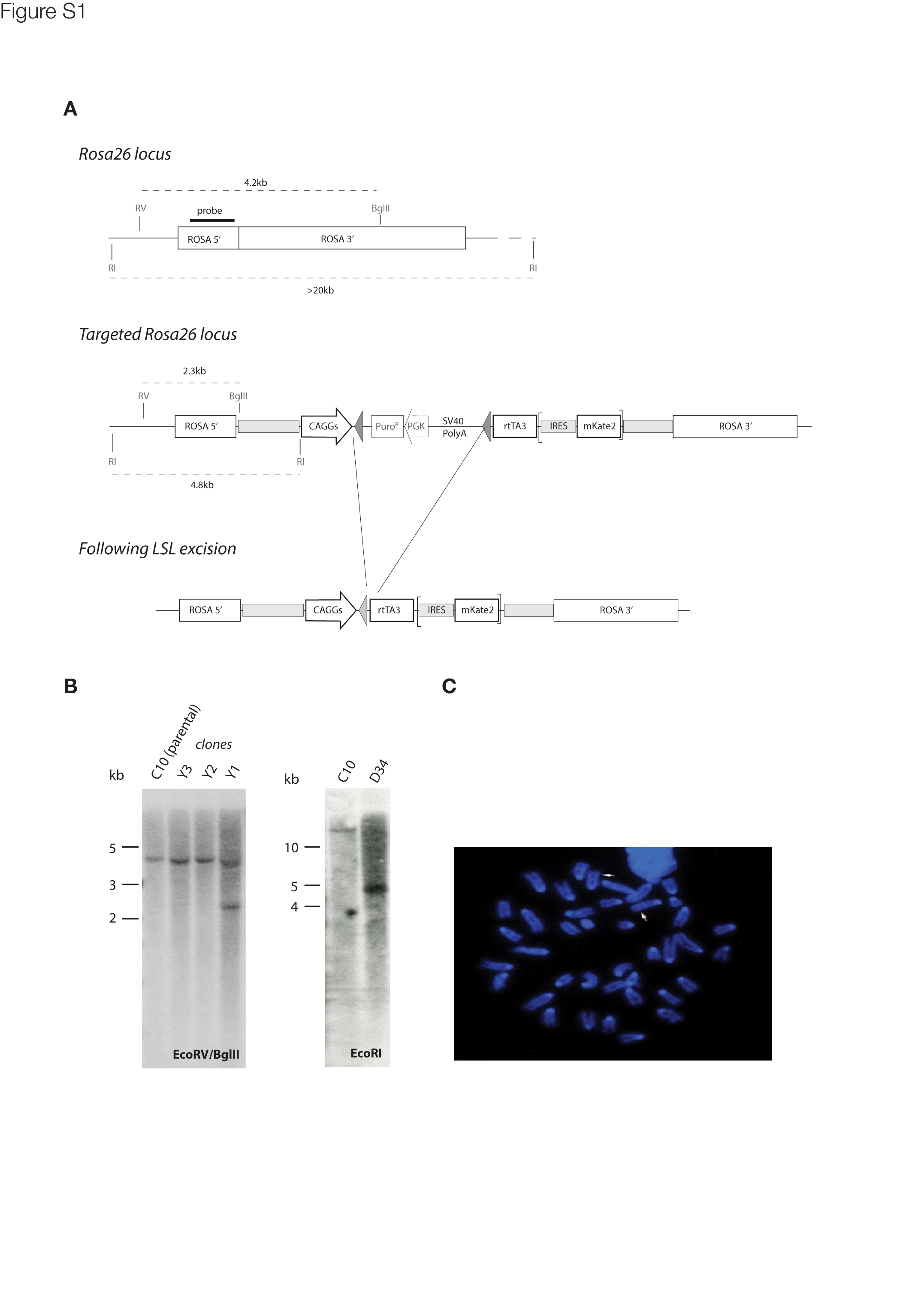

Supplement: Figure S1 — Targeting CAGs-LSL-rtTA3 to the Rosa26 locus. A. Schematic of the Rosa26 locus before and after recombination of the CAGs-LSL-rtTA3 or CAGs-LSL-RIK targeting vector. Key restriction sites used for clone identification by Southern blot are indicated. Sizes of each predicted fragment are also shown and a solid black line highlights the position of the Southern probe. B. Southern blot images showing identification of Y1 (2.3 kb band) and D34 (4.8 kb band) clones, following EcoRV/BglII and EcoRI digests, respectively. C. Fluorescence in situ hybridization on a metaphase spread from D34 ES cells using the CAGs-LSL-RIK fragment as a probe, showing homozygous targeting of CAGs-LSL-RIK to Chromosome 6. (TIF) [file pone.0095236.s001.tif]

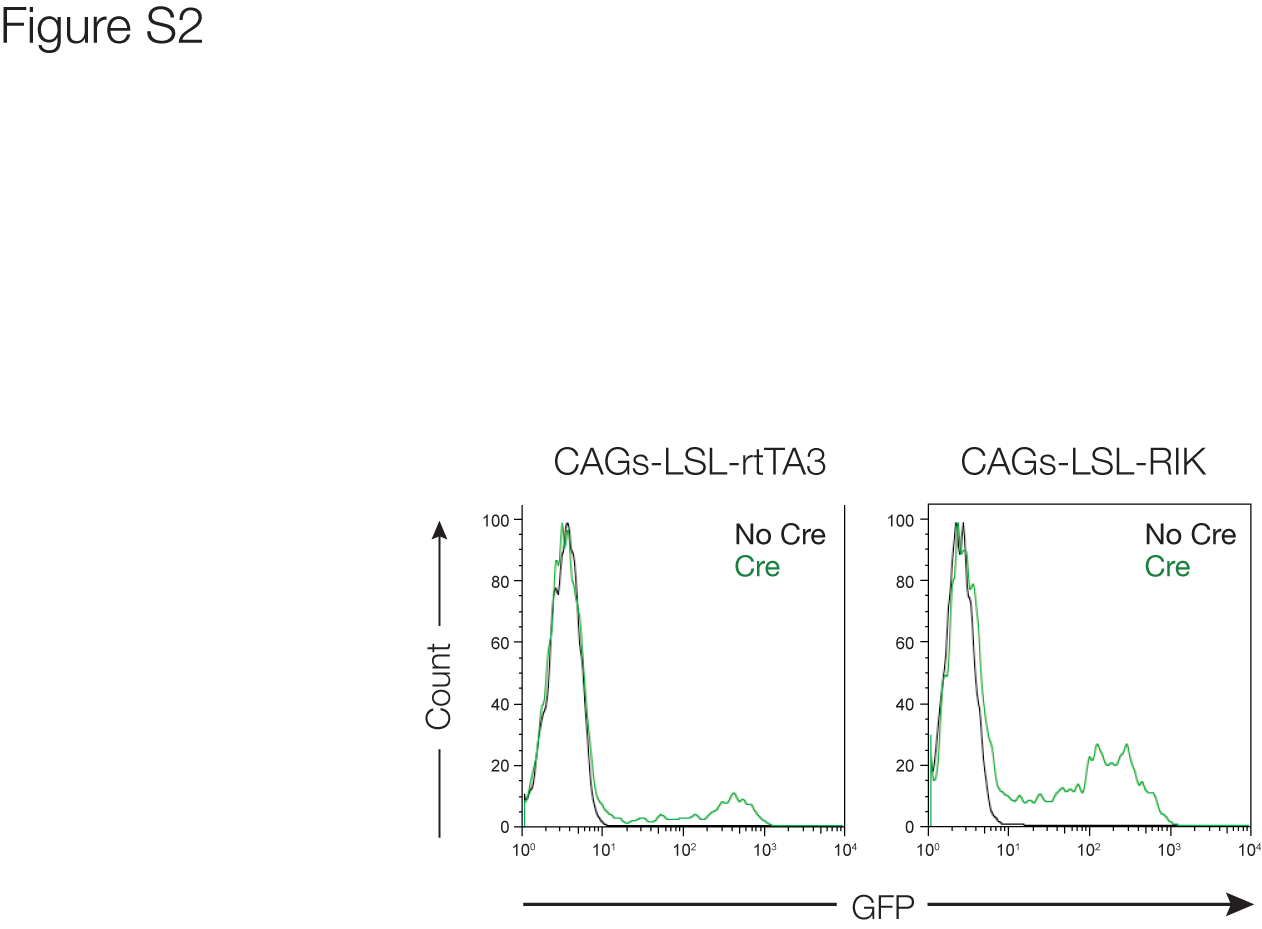

Supplement: Figure S2 — GFP induction following Adenoviral Cre transduction in targeted Y1 and D34 ESCs. Y1 and D34 ESCs carrying TG-Ren.713 at the col1a1 locus were transduced with adenovirus expressing Cre (Cre, green line) or not transduced (no Cre, black line), treated with doxycycline (1 ug/ml) for 2 days and analyzed by flow cytometry. Graphs represent bulk population of transduced cells (not single clones). Bulk populations were single cell cloned to assess the uniformity of GFP induction in the presence of constitutive rtTA3 expression (see Fig. 1B). (TIF) [file pone.0095236.s002.tif]

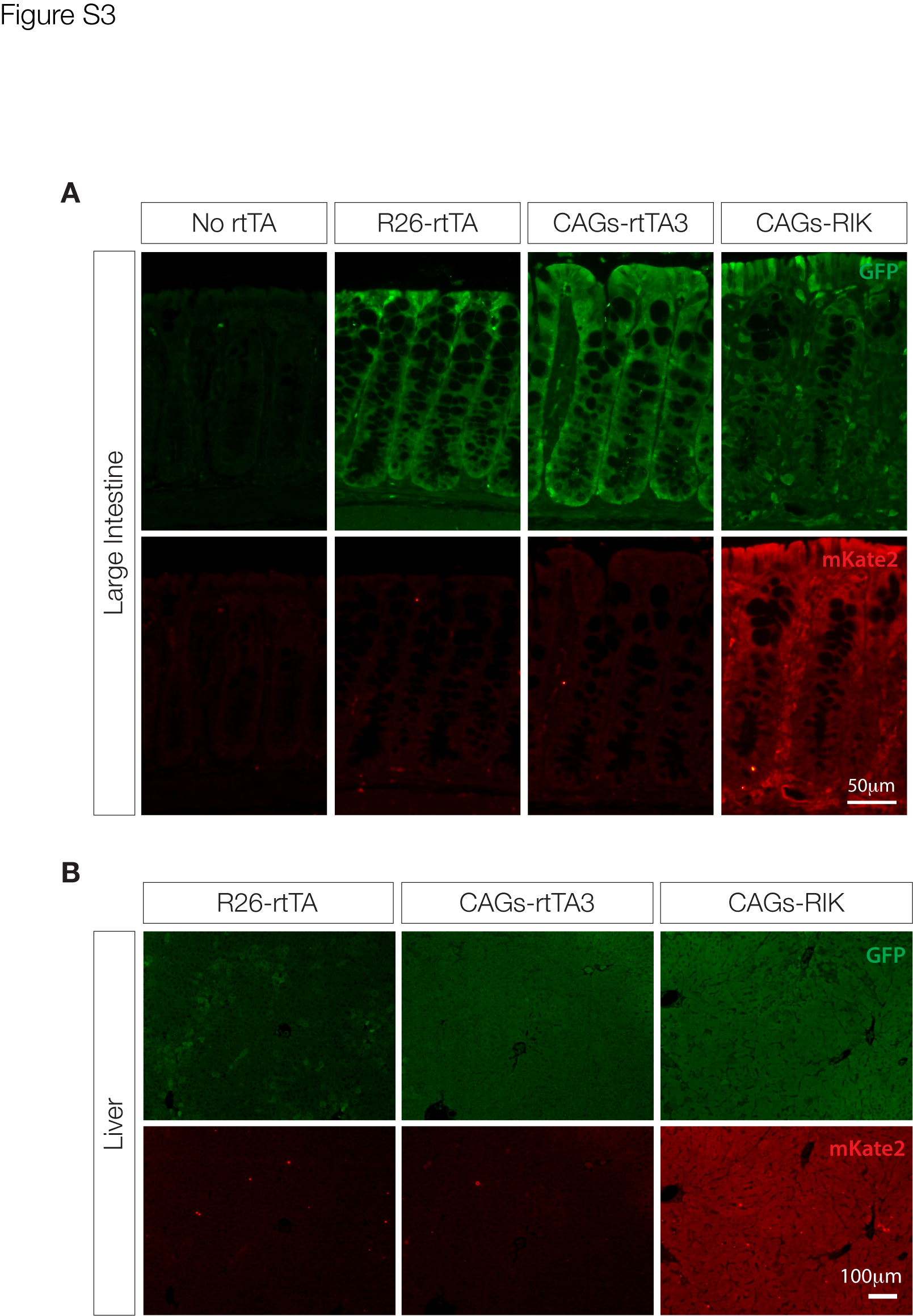

Supplement: Figure S3 — GFP induction and mKate2 expression in large intestine and liver. Immunofluorescence stains for GFP and mKate2 in the large intestine and liver of ‘no rtTA’, R26-rtTA, CAGs-rtTA3 and CAGs-RIK mice following 1 week of doxycycline treatment. All rtTA strains show strong GFP induction in large intestine (A), but only CAGs-rtTA3 and CAGs-RIK show robust and uniform GFP expression (and mKate2 for RIK) in the liver tissue (B). (TIF) [file pone.0095236.s003.tif]

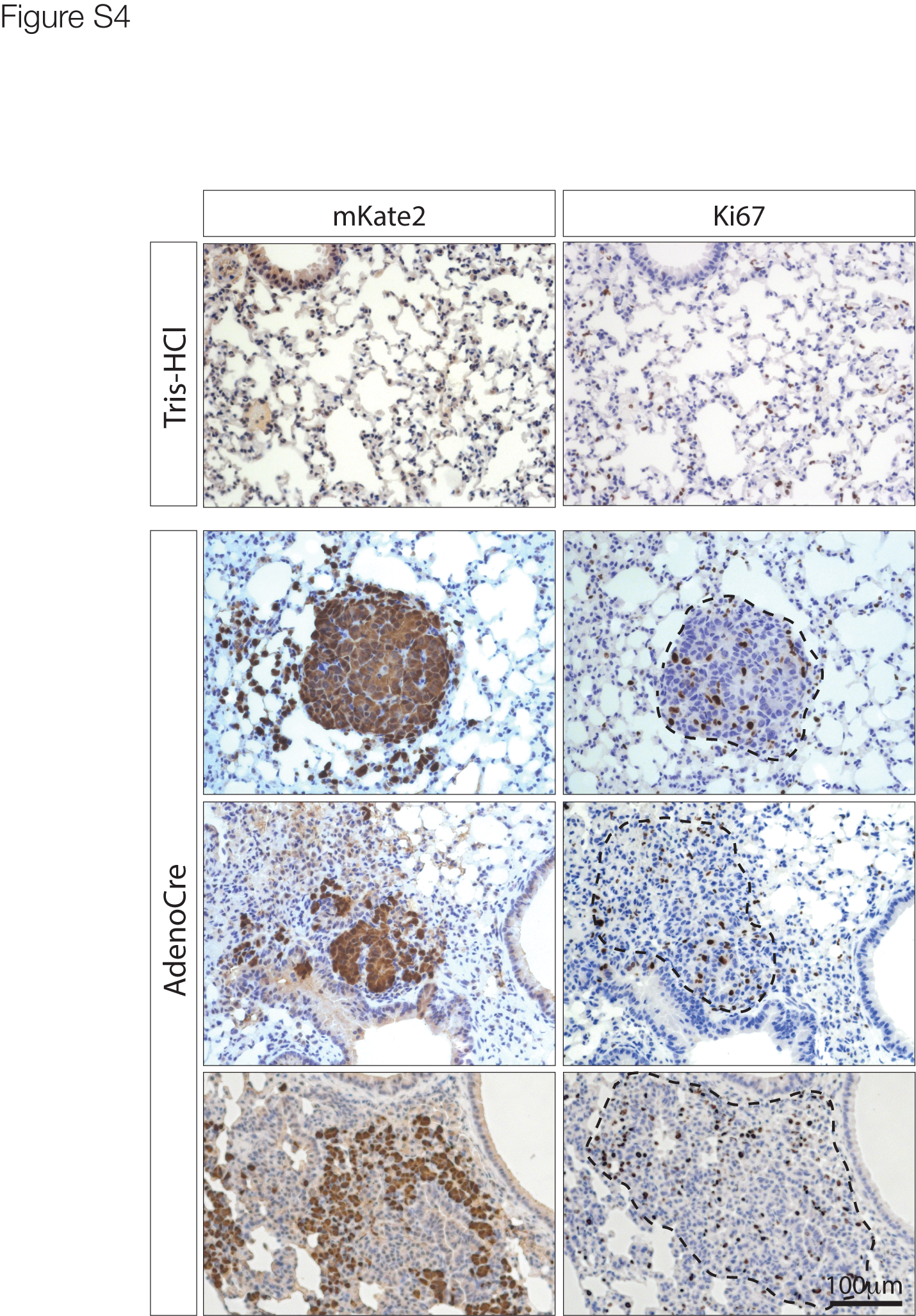

Supplement: Figure S4 — Mosaic mKate2 expression in a proportion of lung adenomas. Immunohistochemical stains for mKate2 and Ki67 in lung sections of double transgenic mice (CAGs-LSL-RIK;LSL-KrasG12D) treated with intratracheal Adenoviral Cre (AdenoCre) or vehicle (Tris-HCl). 12 weeks following Cre delivery LSL-KrasG12D mice show small, moderately proliferative adenomas. Some adenomas show uniform mKate2 staining (top panel of ‘AdenoCre’), while a subset showed both positive and negative mKate2 cells (arrows) suggesting Cre-driven activated KrasG12D but not rtTA3-IRES-mKate2. Adenoma area highlighted by dotted line. (TIF) [file pone.0095236.s004.tif]
