# Supplementary material for: Conditional Reverse Tet-Transactivator Mouse Strains for the Efficient Induction of TRE-Regulated Transgenes in Mice
Source: PLoS One. 2014 Apr 17;9(4):e95236. doi: 10.1371/journal.pone.0095236 (PMC3990578; doi:10.1371/journal.pone.0095236)
Supplement: Table S1 — Genotyping primers. Primer sequences and expected PCR product sizes for genotyping Rosa26-targeted CAGs-rtTA3 strains. (DOCX) [file pone.0095236.s005.docx]

**Table S1: Genotyping primers**

| Genotyping primers | Sequence | Conc (μM) | Expected sizes | Notes |
| --- | --- | --- | --- | --- |
|  |  |  |  |  |
| ROSA A | AAAGTCGCTCTGAGTTGTTAT | 10 | Transgene = ~350bp  Wild-type = ~297bp | Determines wild-type, heterozygous  and homozygous states |
| ROSA B | GCGAAGAGTTTGTCCTCAACC | 10 |  |  |
| ROSA C5 | CCTCCAATTTTACACCTGTTC | 10 |  |  |
|  |  |  |  |  |
| CAGs-rtTA3 F | GTTCGGCTTCTGGCGTGTGA | 10 | CAGs-LSL-rtTA = ~440bp  CAGs-rtTA = ~375bp | Does NOT produce a wild-type band |
| CAGs-rtTA3 R | CGCTTGTTCTTCACGTGCCA | 10 |  |  |
| LSL-rtTA3 F | AAAAACCTCCCACACCTCCC | 10 |  |  |
|  |  |  |  |  |
| mKate2 F | GGTGAGCGAGCTGATTAAGG | 10 | Transgene = ~200bp | Does NOT produce a wild-type band |
| mKate2 R | TTTTGCTGCCGTACATGAAG | 10 |  |  |
